# Artificial Intelligence‐Assisted Treatment Planning in an Interdisciplinary Rehabilitation in the Esthetic Zone

**DOI:** 10.1111/jerd.70034

**Published:** 2025-09-22

**Authors:** F. J. P. O. Fonseca, B. B. R. Matias, P. Pacheco, C. S. A. S. Muraoka, E. V. F. Silva, N. Sesma

**Affiliations:** ^1^ Private Practice Rio de Janeiro Brazil; ^2^ Faculdade São Leopoldo Mandic São Paulo Brazil; ^3^ Department of Prosthodontics, School of Dentistry University of São Paulo (USP) São Paulo Brazil

**Keywords:** aligners, artificial intelligence, CAD‐CAM, dental prosthesis, digital workflow, guided surgery, orthodontics

## Abstract

**Objectives:**

This case report elucidates the application of an integrated digital workflow in which diagnosis, planning, and execution were enhanced by artificial intelligence (AI), enabling an assertive interdisciplinary esthetic‐functional rehabilitation. With AI‐powered software, the sequence from orthodontic treatment to the final rehabilitation achieved high predictability, addressing patient's chief complaints.

**Clinical Considerations:**

A patient presented with a missing maxillary left central incisor (tooth 11) and dissatisfaction with a removable partial denture. Clinical examination revealed a gummy smile, a deviated midline, and a disproportionate mesiodistal space relative to the midline. Initial documentation included photographs, intraoral scanning, and cone‐beam computed tomography of the maxilla. These data were integrated into a digital planning software to create an interdisciplinary plan. This workflow included prosthetically guided orthodontic treatment with aligners, a motivational mockup, guided implant surgery, peri‐implant soft tissue management, and final prosthetic rehabilitation using a CAD/CAM approach.

**Conclusions:**

This digital workflow enhanced communication among the multidisciplinary team and with the patient, ensuring highly predictable esthetic and functional outcomes.

**Clinical Significance:**

Comprehensive digital workflows improve diagnostic accuracy, streamline planning with AI, and facilitate patient understanding. This approach increases patient satisfaction, supports interdisciplinary collaboration, and promotes treatment adherence.

## 1 Introduction

Challenges are inherent in cases requiring dental implants, particularly in esthetic areas. Achieving proper three‐dimensional implant positioning, correct tissue reconstruction, and appropriate prosthetic management are critical for an excellent outcome [1]. To attain these benefits, various methods and tools have been refined to improve reproducibility, predictability, efficiency, and clinical documentation. These factors are largely addressed by incorporating technology into the diagnosis, planning, and execution of dental treatments [2].

The integration of patient's data such as facial scans, high‐resolution tomography, occlusal records with intraoral scans, and photographs combined with digital libraries and customized arch models offers greater speed and precision in steps that were previously more laborious, time‐consuming, and less accurate [3]. The use of digital planning software not only provides faster results but also enhances accuracy, positively impacting the fidelity between the treatment plan through intraoral mockup and the final outcome.

Artificial intelligence (AI) has significantly improved dental treatment planning. AI‐powered software, such as Smile Cloud 3DNA (Straumann Group), expands possibilities beyond integrated planning by creating dynamic, demonstrative videos. Using initial photographic documentation, this software generates a realistic simulation of the proposed treatment, increasing predictability and enhancing the patient's experience while also minimizing dental treatment reworks due to aesthetic dissatisfaction. Similarly, coDiagnostix (Straumann Group), a dental implant planning software, helps to precisely define the optimal placement of the future implant, reducing procedural uncertainties [4, 5, 6].

For the clinicians, it means higher predictability, efficiency, and assertiveness in communication with both the interdisciplinary team and the patient, leading to greater confidence. For the patient, it improves communication and understanding of the treatment plan, leading to a more transparent process and increasing treatment acceptance and engagement [7].

The purpose of this case report is to describe the esthetic‐functional rehabilitation of a patient with a missing central incisor, demonstrating the use of an integrated digital workflow with AI for the efficient diagnosis, planning, and execution of an interdisciplinary treatment, with final results aligned with the initial plan.

## 2 Case Report

**FIGURE 1:**
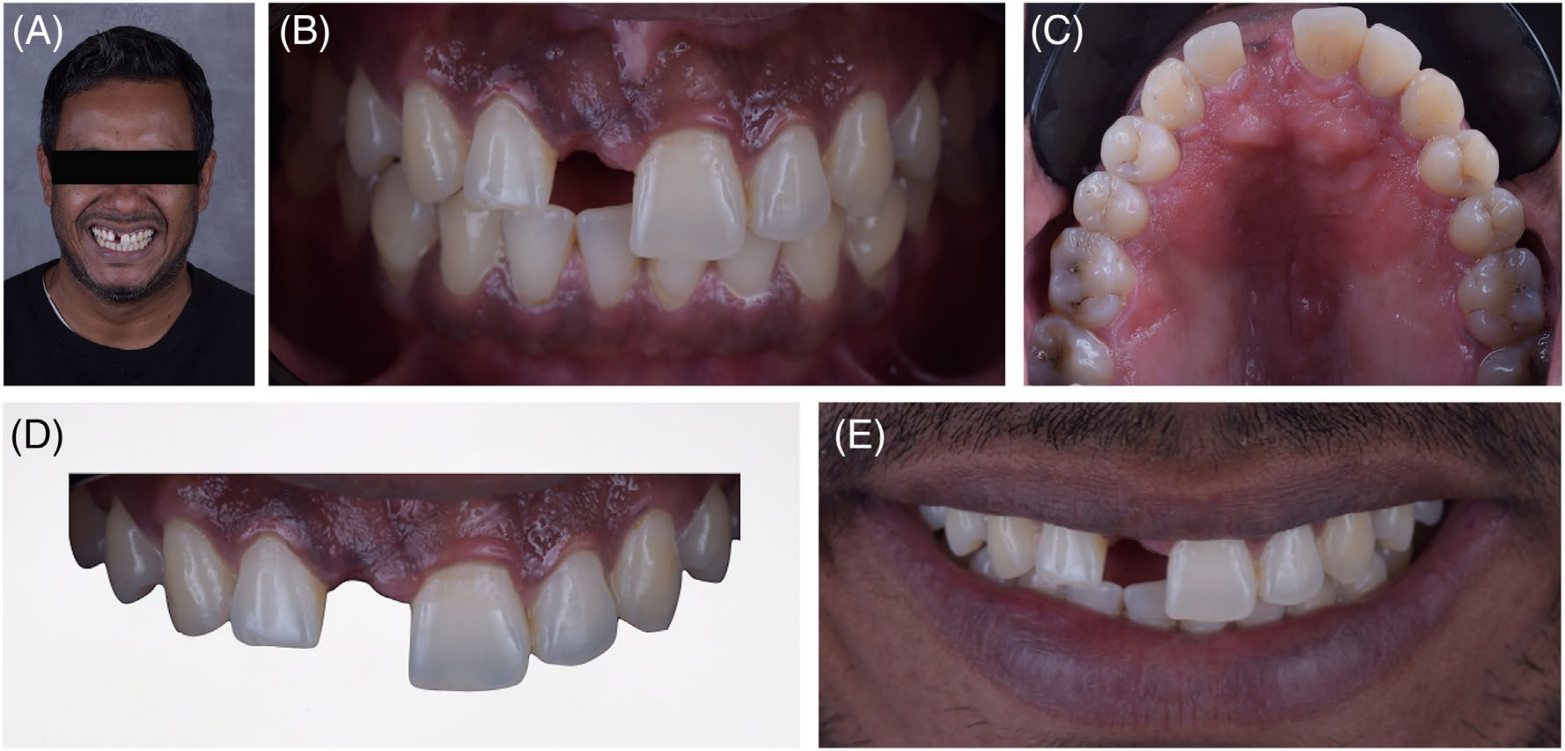
Initial situation. (A) front view of the smile; (B) intraoral view; (C) maxillary occlusal view; (D) upper occlusal view; (E) close‐up photography of the smile.

**FIGURE 2:**
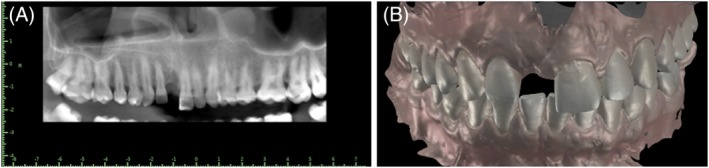
(A) Cone beam computed tomography (DICOM viewer); (B) PLY initial situation.

A 35‐year‐old male patient presented to the clinic with the chief complaint of a missing maxillary left central incisor 11 and dissatisfaction with a removable partial denture. Initial clinical examination revealed a gummy smile, a deviated dental midline, and a disproportionate mesiodistal space relative to the sagittal midline in the anterior maxilla. For an individualized treatment plan, a comprehensive initial documentation was performed, which included intraoral and extraoral clinical photographs (frontal, lateral at rest, and at a maximum smile) (Figure 1), intraoral scans of the dental arches (SIRIOS, Straumann Group), and occlusal records. Additionally, a cone‐beam computed tomography (CBCT) scan of the maxilla was provided (Figure 2). All these data were integrated for a detailed initial analysis, considering the patient's specific complaints considering facial and dental measurements. A written consent was obtained from the patient.

### 2.1 Digital Workflow and AI‐Assisted Esthetic Planning

A reverse planning in a prosthetically‐driven rehabilitation was created using the SmileCloud 3DNA software. In this program, AI analyzes facial and dental features to quickly and objectively suggest personalized esthetic arrangements. The plan incorporated high‐resolution data from the CBCT and intraoral scan, which allowed the measurement of bone dimensions at the site of tooth 11 and the available prosthetic space. This analysis confirmed that implant placement was feasible without the need for prior or simultaneous bone reconstruction [8]. However, it was also noted that there was insufficient mesiodistal space for a prosthetic crown to be in harmony with the contralateral tooth 21.

**FIGURE 3:**
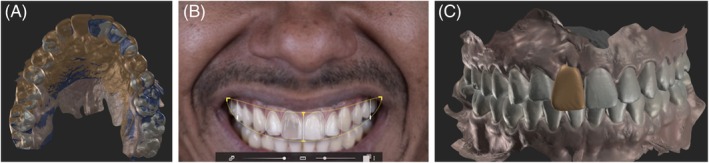
Initial esthetic planning simulation in software (SmileCloud 3DNA).

**FIGURE 4:**
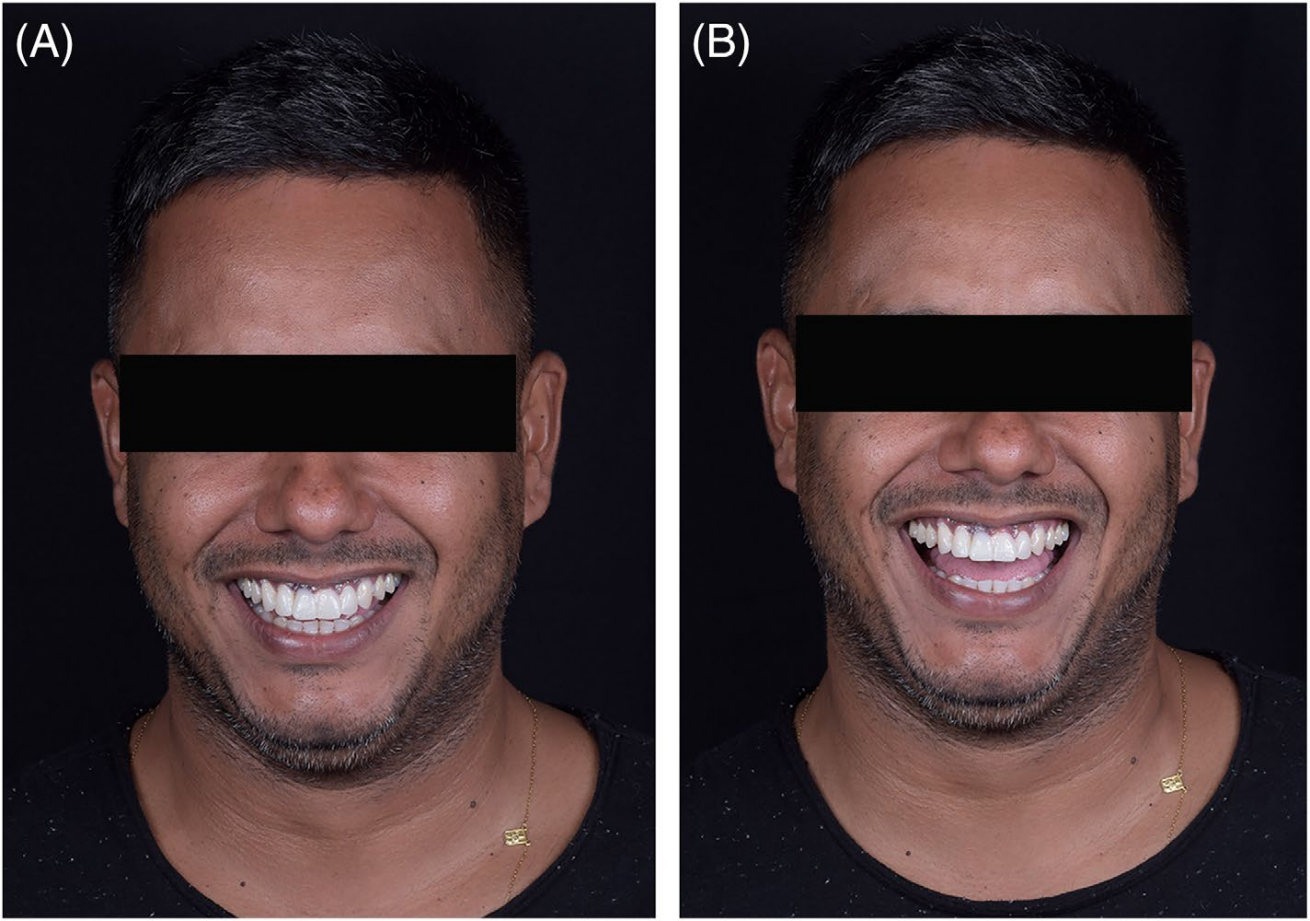
Motivational mockup during a spontaneous smile (left) and at maximum smile (right).

To enhance communication and visualization of the final rehabilitation, a model with the diagnostic wax‐up from the SmileCloud 3DNA software was printed (Figure 3 and Annex S1). A silicone matrix was fabricated from this model and then loaded with bis‐acrylic resin to create a motivational mockup on teeth 12 through 22 (Figure 4). This esthetic trial validated the plan through a realistic and tangible visualization, ensuring mutual agreement between the dentist and the patient.

### 2.2 Prosthetically Guided Orthodontic Treatment

**FIGURE 5:**
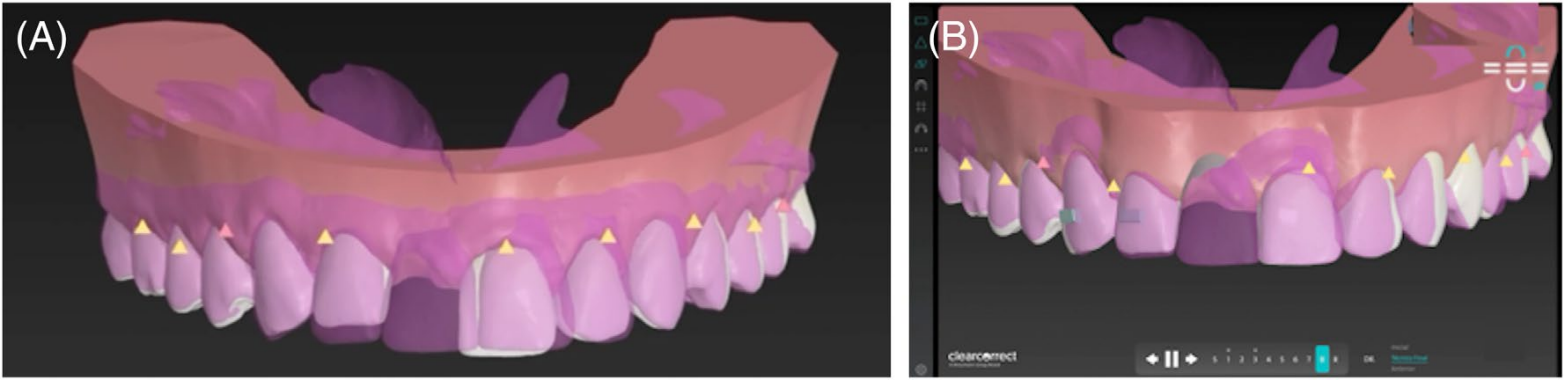
A digital workflow for orthodontic treatment using ClearCorrect. (A) Initial situation; (B) Simulation of the expected result after treatment with aligners.

Based on the high smile line, deviated midline, and inadequate mesiodistal space in the anterior region, orthodontic treatment with aligners (ClearCorrect, Straumann Group) was indicated. The final prosthetic planning (Figure 5 and Annex S2) guided the entire orthodontic movement. This involved distalization, arch perimeter expansion, and correction of the dental midline relative to the facial midline through interproximal reduction (IPR) and strategic placement of attachments.

The aligners were designed to achieve the ideal tooth arrangement established in the SmileCloud 3DNA reverse planning, aiming to optimize the space for the implant and correct the mesialization of teeth 21–27, creating harmonious spacing between the dental arches. The patient received instructions for the continuous, daily wear of the aligners as prescribed by the manufacturer, removing them only for eating and oral hygiene. Specific oral hygiene instructions were also provided [9].

**FIGURE 6:**
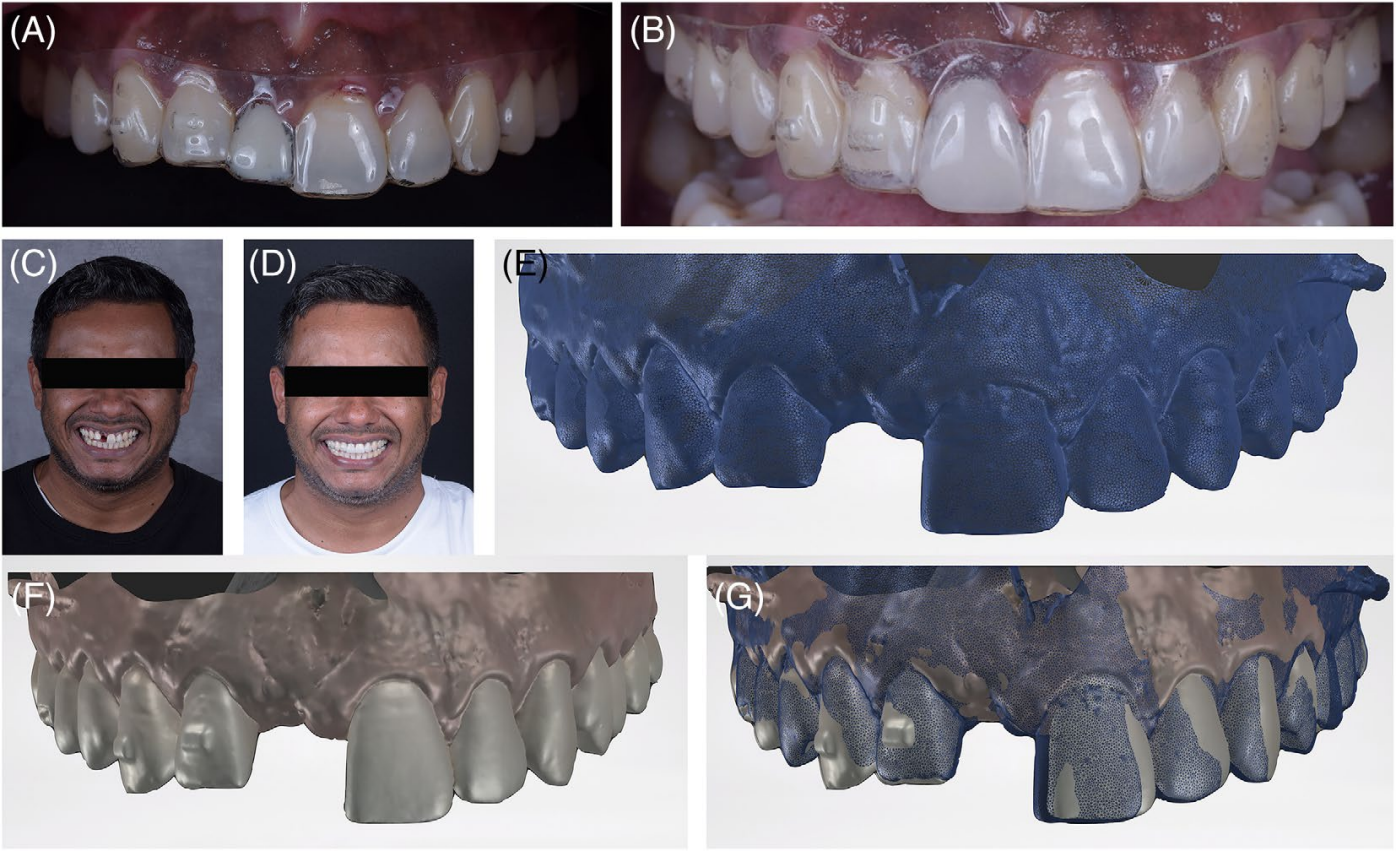
Orthodontic treatment (A) Intraoral view of the aligner showing a limited prosthetic space in the area of tooth 11, with a temporary crown attached to the aligner; (B) intraoral view of the final aligner, with adequate prosthetic space in the area of tooth 11; (C) frontal view of the smile before orthodontic treatment; (D) frontal view of the smile with the last aligner; (E) pre‐orthodontic treatment scan (in blue); (F) post‐orthodontic treatment (in color); (G) superimposition of the initial and final scans.

During treatment, the internal surface of the aligner was provisionally filled with resin in the central incisor region (Figure 6A). The total orthodontic treatment duration was 16 weeks, including eight aligners that were changed progressively every 15 days. The final aligner was adapted as a temporary retainer, now incorporating an acrylic resin tooth in the 11 region with correct prosthetic proportions and in harmony with the contralateral tooth (Figure 6D).

### 2.3 Guided Surgery for Implant Placement and 3D‐Printed Custom Healing Abutment

**FIGURE 7:**
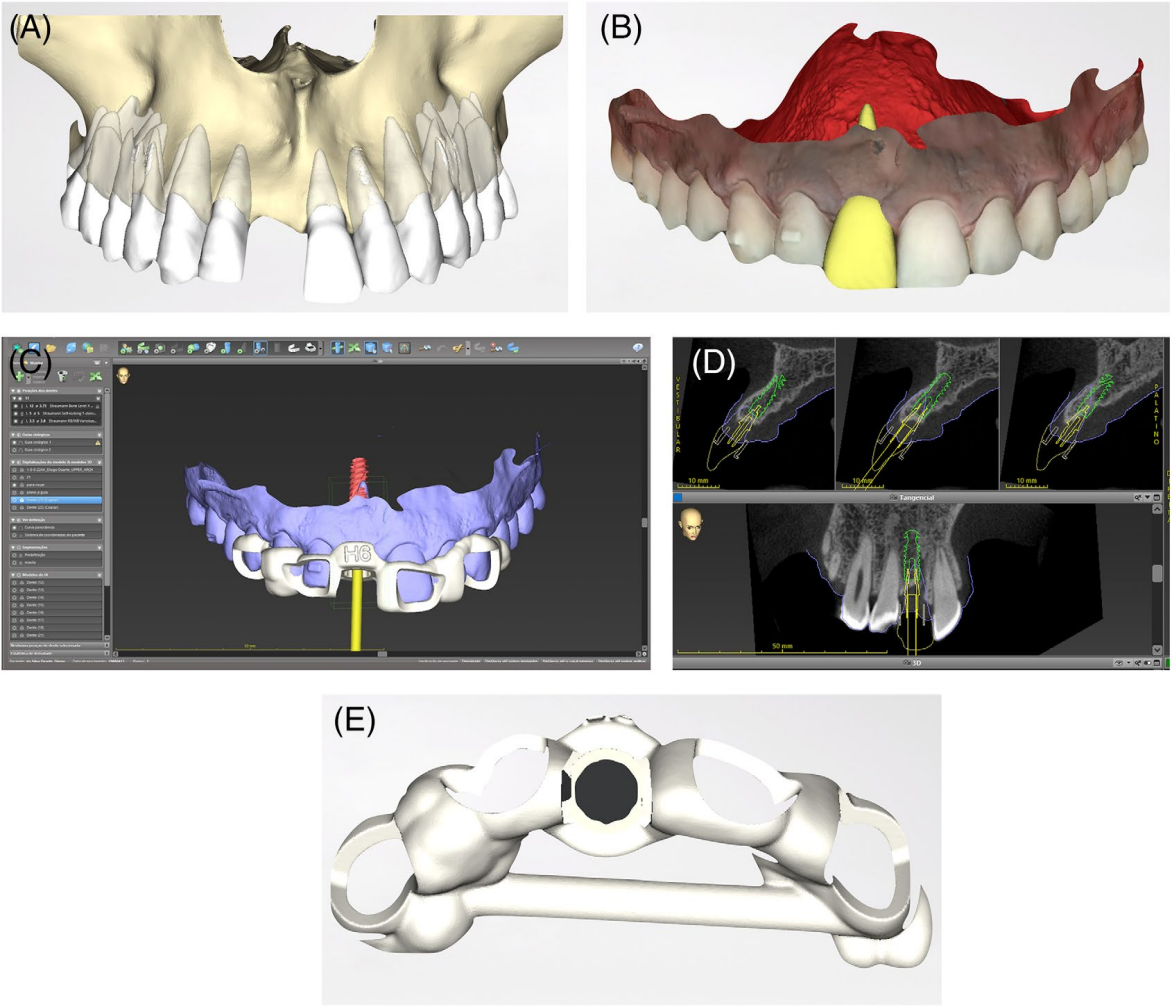
Surgical implant planning. (A) 3D reconstruction of the full maxillary tomography scan with dental segmentation; (B) mirroring of the anatomy of tooth 21 to the site of tooth 11; (C) virtual design of the implant surgical guide in software; (D) Three‐dimensional positioning of the future implant, with crown‐guided planning; (E) the generated .stl file for 3D printing of the surgical guide.

At the end of all orthodontic treatment, a new CBCT and intraoral scan were provided. The DICOM and PLY files were superimposed in the coDiagnostix planning software to precisely plan the future implant position [10]. The surgical guide for the BLX Guided Surgery system (Straumann Group) was 3D printed and pre‐operatively tried‐in to ensure accurate fit and function (Figure 7) [11].

**FIGURE 8:**
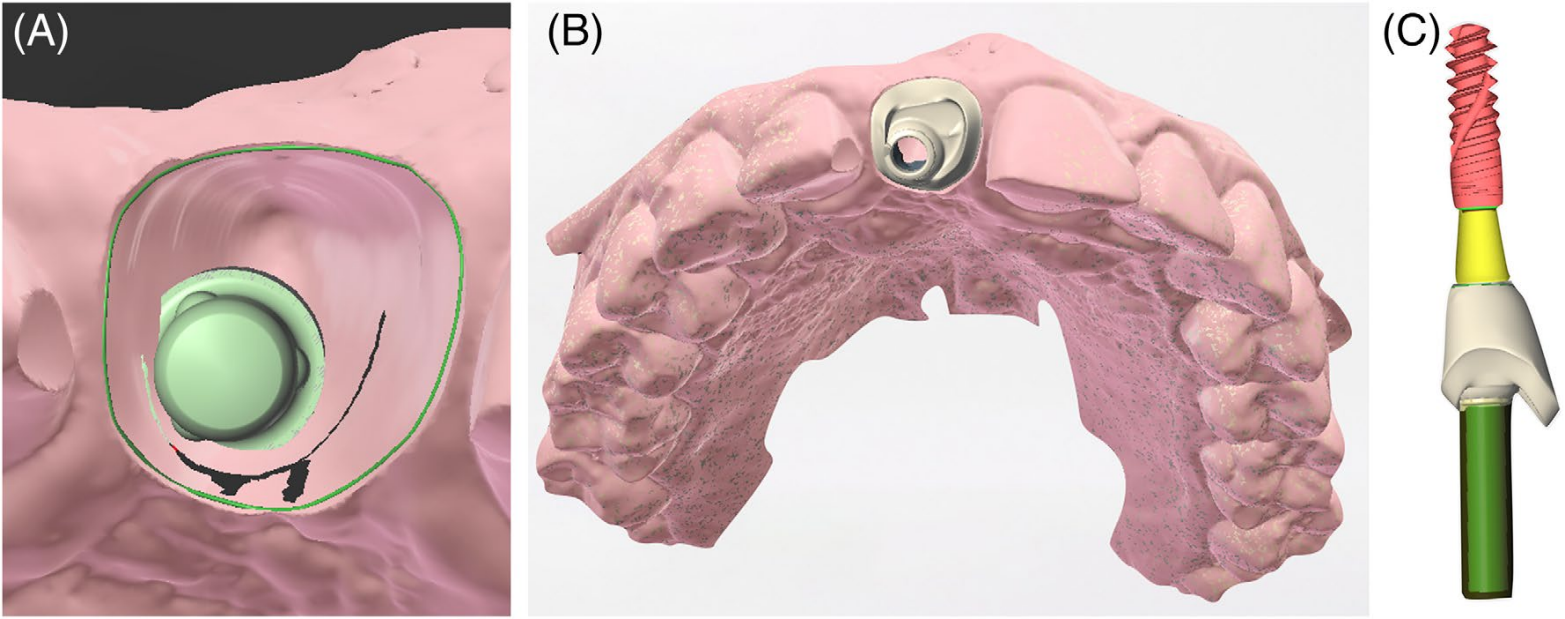
(A) Virtual emergence profile of tooth 11 obtained by mirroring the profile of tooth 21 in coDiagnostix; (B) working cast with a custom healing abutment; (C) virtual assembly of the implant, Variobase, and custom healing abutment.

Virtually, in the Straumann Cares Visual software (Straumann Group), tooth 21 was mirrored to the edentulous site. The portion of the virtual crown corresponding to the critical and subcritical profiles was digitally maintained to design a custom healing abutment (Figure 8). This abutment was 3D printed with Cosmos Temp resin (Yller) on a Spectra printer (Straumann Group) and cemented to a T‐base metal abutment (Straumann Group) with resin cement (3M) prior to surgery. This ensured the development of a correct prosthetic emergence profile and prevented lateral forces from the temporary orthodontic retainer.

**FIGURE 9:**
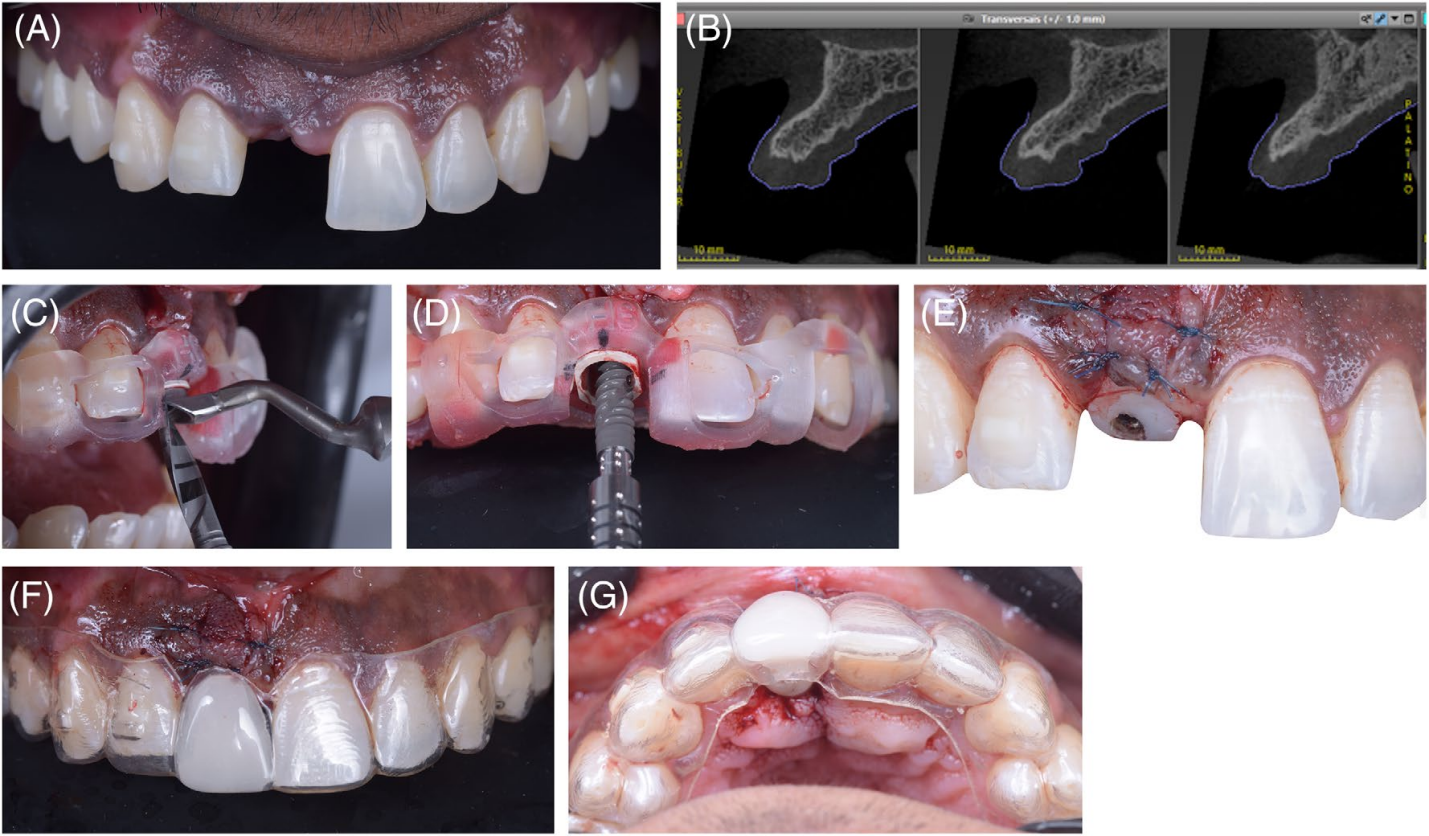
Guided implant surgery. (A) Intraoral view of the surgical site; (B) tomography cross‐section of the 11 region; (C) intraoperative view of the guided instrumentation; (D) dental implant (3.75 × 12 mm BLX) placement using a surgical guide; (E) custom healing abutment; (F) post‐surgical frontal view with the adjusted aligner; (G) occlusal view of the adjusted aligner.

The surgical procedure was performed under local anesthesia in an outpatient setting. The approach involved a flap to preserve the papillae, followed by bone instrumentation and guided placement of an SLActive‐surfaced BLX implant (3.75 mm × 12 mm; Straumann Group) (Figure 9) [12]. Primary stability was measured at 60 N/cm with a surgical torque wrench, and then the custom healing abutment was installed. The orthodontic retainer was then adjusted and repositioned to ensure no contact with the healing abutment (Figure 9). Although the SLActive surface allows implant loading in 21 days, a 60‐day healing period was observed for proper soft tissue maturation [13].

### 2.4 Prosthetic Rehabilitation

**FIGURE 10:**
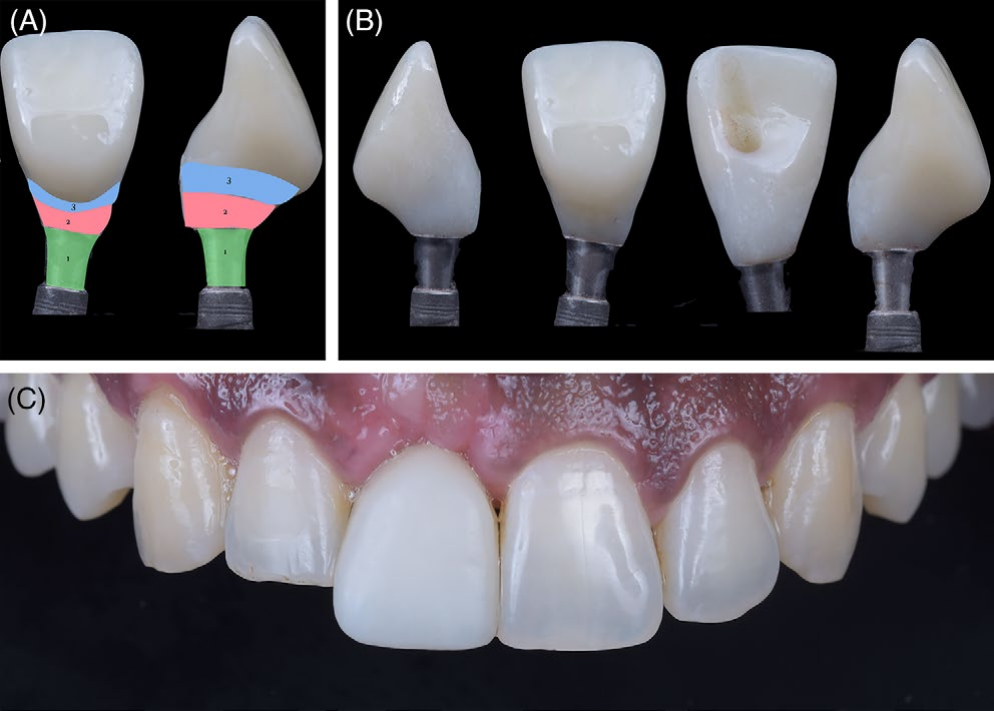
Provisional crown. (A) Schematic drawing of the critical and subcritical profile of the provisional crown; (B) polished and finished provisional crown; (C) provisional crown screwed onto the implant.

A new intraoral scan was performed to capture the accurate gingival emergence profile created by the custom healing abutment, and then a fixed implant‐supported provisional crown was fabricated using 3D printing with PPro Crown and Bridge resin in shade A1 (Straumann Group), which was cemented onto a Variobase metal abutment (Straumann Group). The provisional crown demonstrated a precise emergence profile, proper contours, and excellent polish, encompassing both the critical and subcritical contours (Figure 10).

**FIGURE 11:**
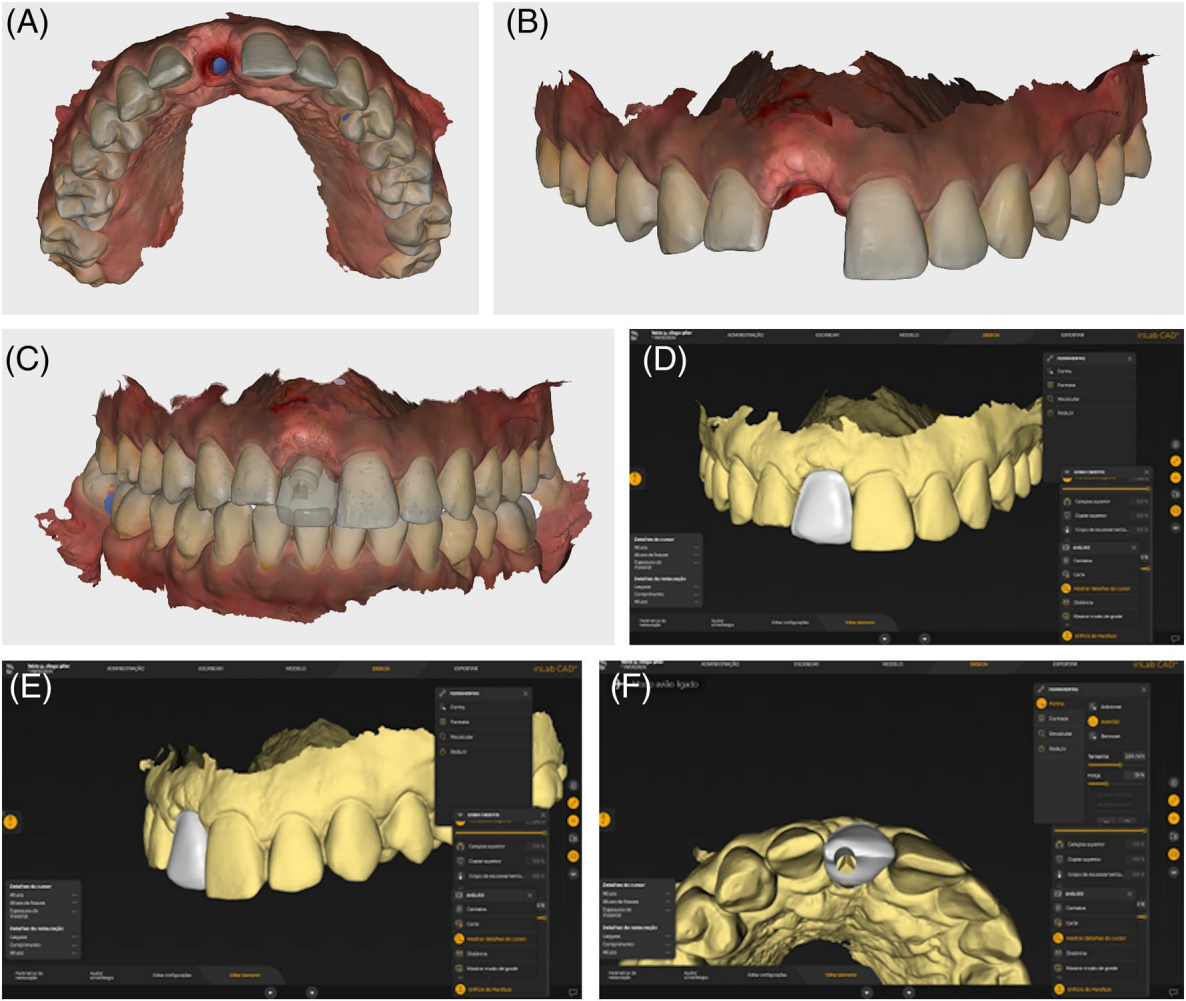
Scanning and CAD of the Custom Abutment for Tooth 11. (A) Occlusal view of the gingival profile obtained with the provisional restoration; (B) buccal view of the gingival profile; (C) alignment of the STLs obtained from the provisional scan, scan body, and gingival profile; (D) buccal view of the custom abutment CAD; (E) side view; (F) occlusal view.

**FIGURE 12:**
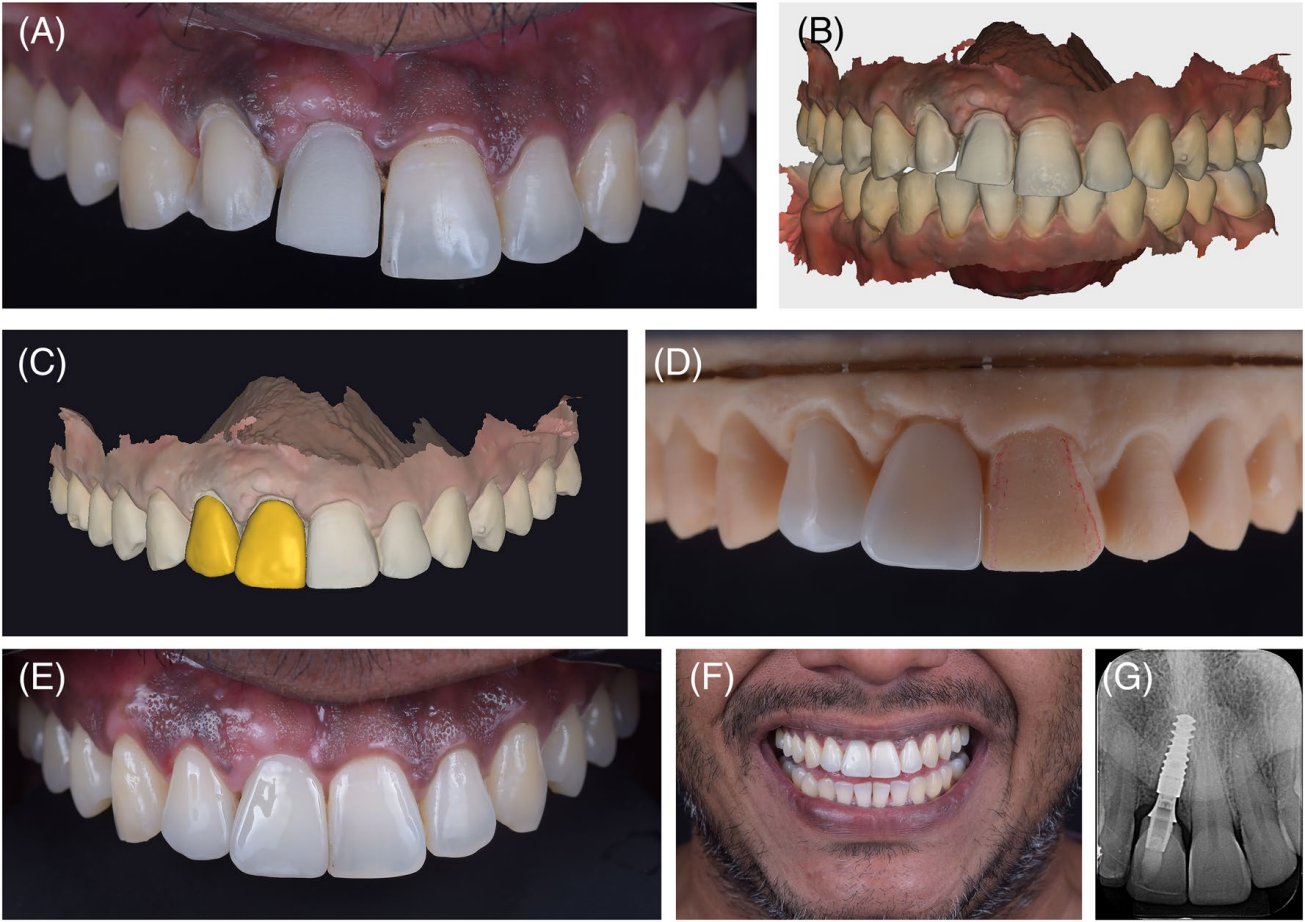
(A) Preparation of tooth 12 for a laminate veneer and try‐in of the custom coping over the Variobase abutment on tooth 11; (B) Scan of the tooth preparation 12 and the custom abutment on tooth 11; (C) CAD of the laminate veneers; (D) ceramic veneers on the model; (E) intraoral view of the laminate veneers; (F) frontal view of the smile with the laminate veneers; (G) periapical radiograph at the completion of treatment.

**FIGURE 13:**
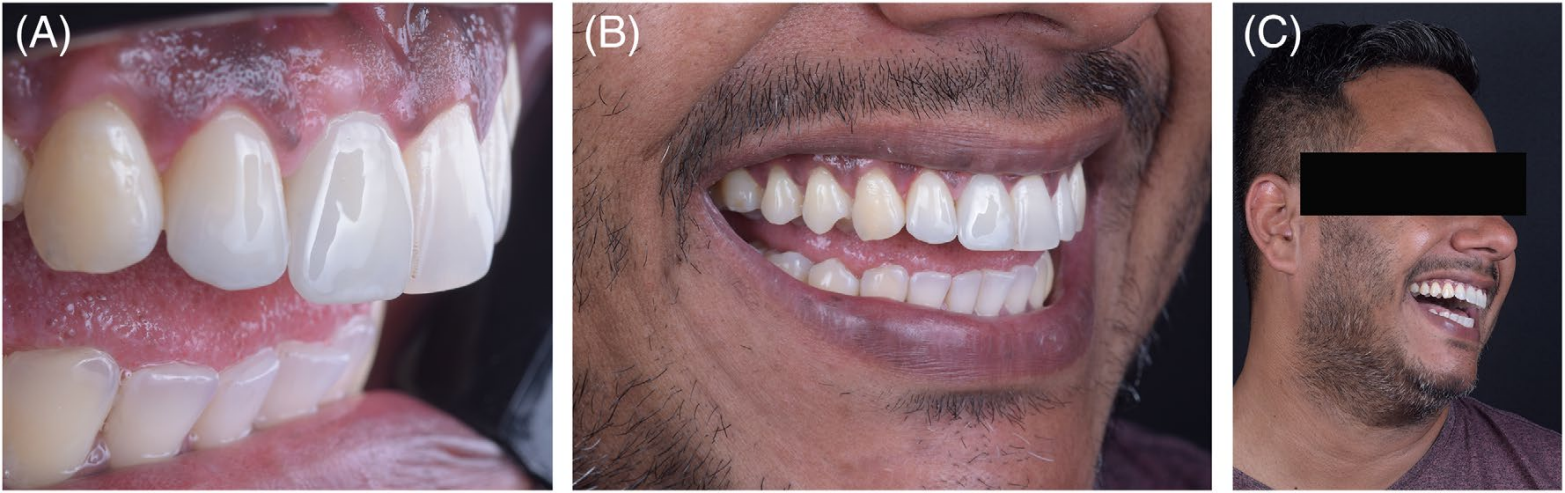
Final clinical photographs. (A) Intraoral lateral view; (B) smile in side view; (C) lateral facial view.

**FIGURE 14:**
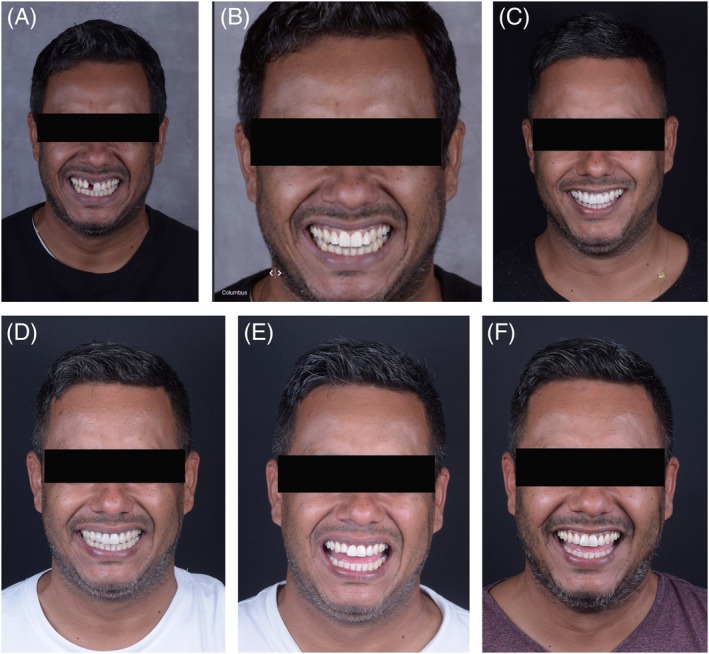
Smile sequence through clinical stages. (A) Initial; (B) esthetic planning in SmileCloud; (C) mock‐up; (D) post‐orthodontic treatment; (E) provisional phase; (F) final prosthetic rehabilitation.

To achieve a harmonious result, a ceramic veneer was planned and fabricated for tooth 12. For the implant at site 11, a custom coping was designed on a Variobase abutment using InLab CAD software (version 22.4.1) (Figure 11) and milled from lithium disilicate in shade A1 (Nice, Straumann Group). The purpose of the custom abutment was to equalize the substrate color of tooth 12 with the coping at site 11. The coping, designed with Exocad software (Align Technology), was also covered with a ceramic veneer (Figure 12) and milled from medium‐translucency lithium disilicate (Emax, Ivoclar) (Figure 13). The use of a digital workflow was crucial to obtain a final esthetic outcome, as it reduced the number of appointments, optimized the process by eliminating the need for conventional impressions, and ensured extreme precision, fit, and accurate contact points, thereby achieving a high degree of esthetic and functional satisfaction (Figure 14) [14].

## 3 Discussion

The complete digital workflow used in this clinical case proved to be highly advantageous compared to conventional methods for diagnosis, planning, and rehabilitation [15, 16]. Unlike traditional techniques that rely on physical impressions, plaster casts, and manual wax‐ups—all of which are susceptible to distortion and require rework—the digital approach allowed more precise, reproducible, and less invasive data acquisition [17]. The integration of data from cone‐beam computed tomography (CBCT), clinical photographs, and 3D scans enabled a detailed three‐dimensional esthetic and functional analysis, facilitating diagnosis and communication among the clinician, the multidisciplinary team, the dental technician, and the patient [16, 17].

The evolution of digital planning not only enhances communication and makes the treatment plan tangible for the patient but also improves communication between the dentist and the laboratory, thereby optimizing clinical outcomes [4, 18]. With proper training, advancements in software allow the dentist to perform laboratory steps such as diagnostic wax‐ups, model printing, and fabrication of guides for esthetic and functional mock‐ups. This strengthens the rehabilitation planning phase and optimizes surgical stages by integrating prosthetic planning, where a lack of integration can lead to errors and compromising outcomes [19].

This clinical case was managed using an integrated workflow with AI‐assisted software to ensure precision, predictability, and personalization at every stage of treatment planning and execution. The process began with facial esthetic planning in the SmileCloud 3DNA software, which created a digital design fully aligned with the patient's individual anatomy and in harmony with the face, along with aligner‐guided orthodontic movement. The incorporation of AI enhanced the accuracy of the esthetic‐functional diagnosis, optimizing clinical decision‐making, interdisciplinary communication, and the patient's understanding of the proposed treatment. Subsequently, coDiagnostix software was used for arch segmentation and mirroring of tooth 21. It not only enabled prosthetically guided implant planning but also generated a mirrored emergence profile, which served as the basis for a custom healing abutment with appropriate dimensions. The final stage involved a new scan, allowing the integration of anatomies in 3DNA for final alignment and case completion, ensuring maximum fidelity between the virtual plan and the clinical result [4, 5, 6].

The digital diagnostic wax‐up and 3D‐printed mock‐up provided a highly accurate simulation of the final result, which improved the patient understanding and acceptance. This approach not only allowed for more assertive pre‐operative adjustments, but also ensured clinical predictability and patient engagement with a fully comprehensible treatment plan—which would not have been entirely feasible without digital resources. In contrast to analog workflows, which require more manual steps and physical communication, the digital environment promoted speed, standardization, and integration among the involved professionals, with a direct impact on treatment efficiency and the quality of the final outcome [20, 21, 22].

The clinical experience presented here reinforces the transformative potential of digital technologies in oral rehabilitation. It is important to note, however, that the success of the treatment still relies on the clinician's clinical foundation and individual esthetic sensibilities.

## 4 Conclusions

This clinical report demonstrates how the strategic adoption of digital resources can transform rehabilitative practice, elevating the standards of precision, predictability, and personalization in dental care. The integration of advanced diagnostics, AI‐assisted digital planning, and technology‐guided execution not only enabled a consistent functional and esthetic outcome but also redefined the patient experience throughout the therapeutic process. When applied with clinical judgment and esthetic sensitivity, digital dentistry establishes itself as a core pillar of contemporary rehabilitation.

## Conflicts of Interest

The authors declare no conflicts of interest.

## Supporting information


**Annex S1:** 2D Simulation video of Digital Smile Design in SmileCloud Software.

**Annex S2:** A simulation video of orthodontic planning in ClearPilot software, guided by the esthetic plan obtained from SmileCloud software.

## Data Availability

The data that support the findings of this study are available on request from the corresponding author. The data are not publicly available due to privacy or ethical restrictions.
